# Status of Treatment and Prophylaxis for Radiation-Induced Oral Mucositis in Patients With Head and Neck Cancer

**DOI:** 10.3389/fonc.2021.642575

**Published:** 2021-03-18

**Authors:** Shiyu Liu, Qin Zhao, Zhuangzhuang Zheng, Zijing Liu, Lingbin Meng, Lihua Dong, Xin Jiang

**Affiliations:** ^1^Department of Radiation Oncology, The First Hospital of Jilin University, Changchun, China; ^2^Jilin Provincial Key Laboratory of Radiation Oncology & Therapy, The First Hospital of Jilin University, Changchun, China; ^3^National Health Commission (NHC) Key Laboratory of Radiobiology, School of Public Health, Jilin University, Changchun, China; ^4^Department of Hematology and Medical Oncology, Moffitt Cancer Center, Tampa, FL, United States

**Keywords:** radiotherapy, oral mucositis, epidemiology, pathogenesis, treatment, prevention

## Abstract

Radiation-induced oral mucositis (RIOM) is one of the most frequent complications in head and neck cancer (HNC) patients undergoing radiotherapy (RT). It is a type of mucosal injury associated with severe pain, dysphagia, and other symptoms, which leads to the interruption of RT and other treatments. Factors affecting RIOM include individual characteristics of HNC patients, concurrent chemoradiation therapy, and RT regimen, among others. The pathogenesis of RIOM is not yet fully understood; however, the release of inflammatory transmitters plays an important role in the occurrence and development of RIOM. The five biological stages, including initiation, primary damage response, signal amplification, ulceration, and healing, are widely used to describe the pathophysiology of RIOM. Moreover, RIOM has a dismal outcome with limited treatment options. This review will discuss the epidemiology, pathogenesis, clinical appearance, symptomatic treatments, and preventive measures related to this disease. We hope to provide a reference for the clinical treatment and prevention of RIOM in HNC patients after RT.

## Introduction

Head and neck cancer (HNC) is a common type of neoplasm, including neck tumors, otolaryngology tumors, and oral-maxillofacial tumors, such as nasopharyngeal, oropharyngeal, hypopharyngeal, and laryngeal cancers. In recent years, radiotherapy (RT) has become increasingly popular as a treatment for HNC patients. RT techniques include intensity-modulated radiotherapy (IMRT), stereotactic body radiation therapy, particle therapy, and high-dose-rate brachytherapy (1). The typical radiation regimen for HNC patients comprises a dose of 2 Gy per day for 5–7 continuous weeks, with a total cumulative dose of 60–70 Gy (2). Due to the relationship between the primary site of HNC and the occurrence of cervical lymph node metastasis, the oral mucosa inevitably accounts for a part or all of the target area in HNC patients undergoing RT; thus, it is exposed to a certain dose of irradiation. Radiation-induced oral mucositis (RIOM) represents a major complication in HNC patients undergoing RT, occurring in almost all patients treated for cancers of the mouth, oropharynx, and nasopharynx. RIOM is an inflammatory or ulcerative lesion caused by radiation-induced damage to basal cells rather than direct damage to superficial cells (3). In this review, we will discuss some recent topics dealing with the epidemiology, mechanisms, and clinical manifestations of RIOM and various approaches for the prevention and treatment of mucositis related to RT.

## RIOM Epidemiology

### Influence Factor of RIOM

RIOM is found in virtually all HNC patients who undergo RT, with the incidence exceeding 90% in patients treated with standard regimens (4). A study has shown that HNC patients with nasopharyngeal or oropharyngeal tumors and those who receive cumulative radiation doses >5000 cGy or concurrent chemoradiation therapy (CCRT) are more likely to develop RIOM (5).

### Self-Related Factors in HNC Patients

Several patient-related factors, such as age, weight, sex, nutritional status, oral microbiota, and oral health status, have been identified as risk factors associated with RIOM development (6, 7). Poor oral health habits, smoking, and malnutrition all result in an increased incidence of RIOM (5).

### Chemotherapy

The incidence of RIOM increases with the use of concurrent chemotherapy. HNC patients with CCRT present improved local tumor control at the expense of increased risk of RIOM. Chen et al. (8) explored the changes in the prevalence of severe RIOM and RIOM-related symptoms over an 8-week period. Their findings showed that HNC patients had a higher prevalence of RIOM when treated with combined RT and chemotherapy than when treated with RT alone. Elting et al. (4) reported that RIOM was more common in patients receiving chemotherapy combined with IMRT (OR = 7.8) than in patients receiving IMRT alone. A phase 3 multicenter randomized controlled trial (NCT00677118) evaluated the efficacy of adjuvant chemotherapy. In this study, during CCRT, 31% of patients in the CCRT plus adjuvant chemotherapy group developed RIOM. Noticeably, 21% of patients developed RIOM during the adjuvant chemotherapy period (9). In summary, these studies showed that chemotherapy increased the incidence of RIOM.

### Radiotherapy Regimen

Changes in the dose fractionation protocol and difference in RT techniques result in different incidences of RIOM. Trotti et al. (10) reported a trend for high incidence of Grade III or higher mucositis. In a study of HNC patients treated with altered fractionation RT (1.25–2.00 Gy/f) found that 56% of patients experienced RIOM (Grade III–IV) compared to 34% of patients who received conventional RT. Hsiung et al. (11) evaluated the radiation dose supplied to the oral mucosa during IMRT for HNC and reported that IMRT can reduce the severity of RIOM compared to conventional RT. Romesser et al. (12) randomly assigned 23 HNC patients to be treated with IMRT and 18 HNC patients to be treated with proton beam radiation therapy (PBRT). This study demonstrated that the incidence of RIOM after PBRT was significantly lower than that after IMRT (16.7 vs. 52.2%).

The dose of RT is another factor that affects RIOM. Radiation causes necrosis of the epithelium, leading to desquamation and ulceration. And with the increasing irradiation dose, the more severe of the degree of RIOM. A cumulative radiation dose ≥50 Gy is found to increase the risk of RIOM; when the cumulative radiation dose is ≥65 Gy, the risk of RIOM in HNC patients is the highest (5). Narayan et al. (13) conducted a clinical trial to correlate oral cavity dose with RIOM. And they found that the cumulative point doses < 32 Gy occurred mild severity (Grade </= 1) and short duration (</= 1 week) of mucositis. They also concluded that a dose > 39 Gy was associated with longer duration of mucositis. In a conclusion, limiting dose to < 39 Gy or an average oral mucosa dose < 32 Gy resulted in mild severity and only a short duration of RIOM for HNC patients. Based on this conclusion, Wang et al. (14) made a prospective and comparative trial to observe the incidence of RIOM for HNC patients (*n* = 24) received < 32Gy. And they found that just 25% of HNC patients suffered Grade III RIOM, and they rarely use analgesics and intravenous antibiotics.

### Cetuximab

Comprehensive treatment other than chemotherapy combined with RT may affect RIOM. In the current study, a high incidence of Grade III–IV RIOM was observed in HNC patients receiving RT combined with cetuximab (15). In another study, compared to CCRT alone, adding cetuximab resulted in a reduction in Grade III–IV RIOM incidence (51.6 vs. 23.4%; *P* < 0.001) (16). But some researches detected that cetuximab combinded with RT does not have a significant impact on the incidence of high-grade (≥grade 3) mucositis in comparison to RT alone (17, 18). Bonner et al. (19) had made a phase III randomized trial, HNC patients were randomly assigned to receive RT with or without cetuximab. Five-year overall survival was 45.6% in the cetuximab-plus-RT group and 36.4% in the RT group. However, the incidence of RIOM (93.3 vs. 93.9%) was similar in both groups. In another research, Tejwani et al. (20) found that, when the combination of RT plus cetuximab was compared with radiation alone, the risk ratio for mucositis it was 1.76 (95% CI, 1.5–2.0; *P* < 0.001), suggesting that there was an increased risk of dermatologic toxicities with the combined regimen. Mei et al. (21) searched relevant articles to compare the efficacy of concurrent cetuximab with RT(ExRT) vs. cetuximab combined with RT and chemotherapy (ChRT) in treating HNC patients. And they deduced that cetuximab was an effective radiosensitizer, while ChRT achieved better survival outcomes than ExRT. Additionally, cetuximab combined with RT presented increasing occurrence of mucositis (RR: 1.17, *p* < 0.005) in comparison to ChRT group.

## Pathogenesis

The pathogenesis of RIOM includes both direct and indirect mechanisms. The direct effect is due to DNA strand breakage and apoptosis caused by radiation, resulting in a reduction in the renewal of the basal epithelium (22). The indirect effect is due to factors such as the release of inflammatory transmitters, secretion of salivary glands, and neutropenia, causing the destruction of the oral mucosa (23). At present, the five biological stages proposed by Sonis are widely used to describe the occurrence and development of RIOM (24). The five stages comprise initiation, primary damage response, signal amplification, ulceration, and healing.

### Initiation

The initiation stage of oral mucosal injury occurs rapidly after the administration of radiation and involves DNA and non-DNA damage and the production of reactive oxygen species (ROS). RT directly damages the DNA, resulting in double-strand breaks and apoptosis of basal epithelial cells and submucosal cells. ROS are crucial mediators of downstream biological agents that are released from the epithelium and tissue macrophages. ROS generated by intracellular water ionization cause a series of damages to cells, leading to organelle damage. Mitochondria release additional ROS, which in turn damage cell membranes and connective tissue, stimulate macrophages, and activate molecules of the immunoinflammatory response. The inflammatory substances and pathways released include intracellular proinflammatory chemoradiation associated molecular patterns; intracellular enzymes (lysosomial), which activate extracellular proinflammatory damage associated molecular patterns; altered redox state of the injured tissue; presynthesised interleukins (IL-1α, IL-33); released intracellular hidden antigens which activate complement via antibodies (25). In addition, oxidative stress and ROS production can directly damage cells, tissues, and blood vessels and stimulate the production of a large number of transcription factors, such as NF-κB (26).

### Primary Damage Response

Radiation leads to double-strand DNA breaks and activates many downstream signal transduction pathways. Song et al. (27) summarized fourteen pathways as being most relevant to the development of RIOM, including nitrogen metabolism; Toll-like receptor signaling; NF-κB signaling; B Cell receptor signaling; P13K/AKT signaling; Cell Cycle: G2/M DNA Damage checkpoint receptor; P38 MAPK signaling; Wnt/B-catenin signaling; Glutamate receptor signaling; Integrin signaling; VEGF signaling; IL-6 signaling; Death receptor signaling; SAPK/JNK signaling. A variety of transcription factors are activated, such as p53 and NF-κB. Among them, NF-κB has been suggested to be the most significant transcription factor, and it is related to both toxicity and resistance of tumors to therapy (28). The activation of NF-κB in the nucleus promotes the proliferation of proinflammatory factors, such as interleukin (IL)-1β, IL-6, and tumor necrosis factor-α (TNF-α). These compounds induce cell damage, leading to apoptosis (29). In addition, fibronectin breakdown occurs at this stage. Importantly, all of these changes occur in all cells and tissues that form the mucosa and not just those that form the epithelium.

### Signal Amplification

Some of these proinflammatory cytokines not only damage tissue, but also provide a positive feedback loop to amplify the primary damage response induced by radiation. TNF-α is an efficient activator of NF-κB and sphingomyelinase. TNF-α activates NF-κB and sphingomyelinase activity in the mucosa, leading to more cell death. TNF-α amplify the original signal or activate NF-κB, leading to the initiation of MAPK and COX-2 transcription. The MAPK pathway ultimately results in the activation of caspase 3 and cell death (30). The increased level of sphingomyelinase in the tissue amplifies pro-apoptotic signals that are mediated by the ceramide pathway, promoting the cell death. Both TNF-α and IL-1β induce matrix metalloproteinase (MMP)-1 and MMP-3 activation, and COX-2 initiates and transmits signals that activate MMP-1 and MMP-3, leading to disruption of oral mucosal integrity (31). Many proteins produced during the primary injury response accumulate and target mucosal tissues acting both intracellularly and intercellularly, triggering feedback mechanisms via neuronal and blood flow networks, causing an increasing serum levels of NF-κB, TNF-α, IL-1, and IL-6, which generated abscopal effects and toxicity (32). Abscopal effects and toxicity include sepsis causing systemic inflammatory responses, alterations in body temperature and metabolism, fatigue, and others (33).

### Ulceration

**Figure 1:**
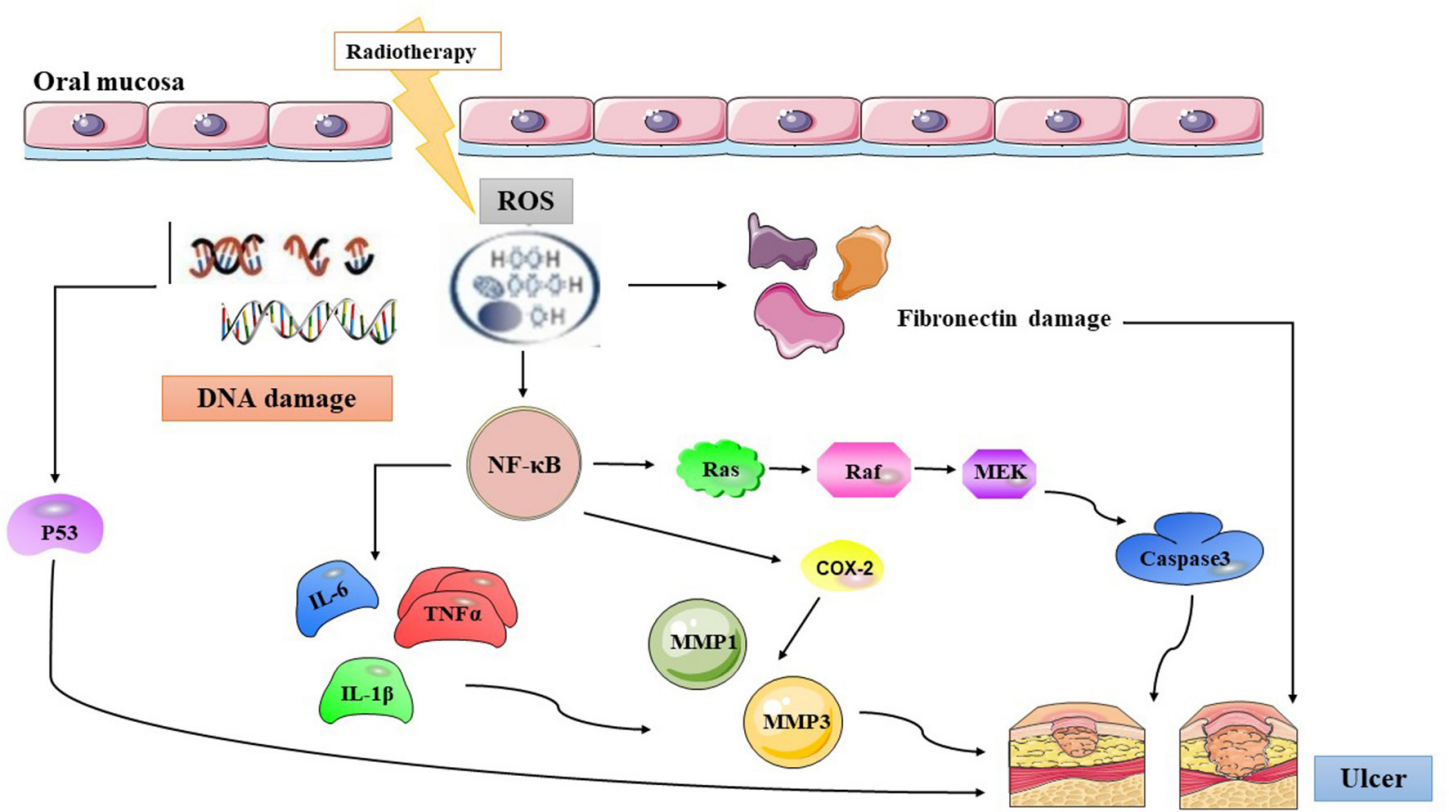
The summary of radiation-induced oral mucositis pathogenesis. Radiotherapy results in direct and lethal DNA damage and releases reactive oxygen species (ROS) from epithelial and tissue macrophages in initiation phase. In primary damage phase, the DNA damage and ROS lead to three major steps: (1) fibronectin breakdown (2) P53 activation (3) nuclear factor-κB (NF-κB) activation that stimulates to release pro-inflammatory cytokines, such as: TNF-α, interleukin (IL)-1β, and IL-6. In the signal amplification phase, NF-κB stimulates the transcription of MAPK, COX-2, etc. The pathway of MAPK actives caspase3, and the other cytokines transmit signals that activate MMP1 and MMP3. Then the pseudomembrane or ulceration appear after around two weeks undergoing with symptomatic treatment of RIOM, and secondary infection adds more pro-inflammatory reactions. *ROS, Reactive Oxygen Species; NF-*κ*B, Nuclear factor kappa-B; IL-6, Interleukin-6; TNF*α*, Tumor Necrosis Factor alpha; IL-1*β*, Interleukin-1*β*; MMP 1, Matrix metalloproteinases 1; MMP 3, Matrix metalloproteinases 3; COX-2, Cyclooxegenase-2*.

During this period, the oral mucosa usually presents with a pseudomembrane or ulceration. This is due to damage to the mucosa. Nerve endings are exposed to the surface, resulting in pain and other symptoms. Microbial colonies appear on the mucosal surface, and cell wall products from the colonizing bacteria are likely to penetrate the submucosa, destroying any new tissue and increasing the release of inflammatory mediators from monocytes (34). This chain of events probably promotes the expression of pro-apoptotic genes and potentiates tissue injury. The pathogenetic characteristics of RIOM in HNC patients are depicted in Figure 1.

### Healing

At this stage, epithelial cell proliferation and histiocyte differentiation can be seen, thereby restoring tissue integrity. Factors affecting the speed of mucosal repair include the rate of epithelial cell migration, rate of proliferation, differentiation of healing tissue, agents selected, and dose and timing of therapy.

## Clinical Appearance

**Figure 2:**
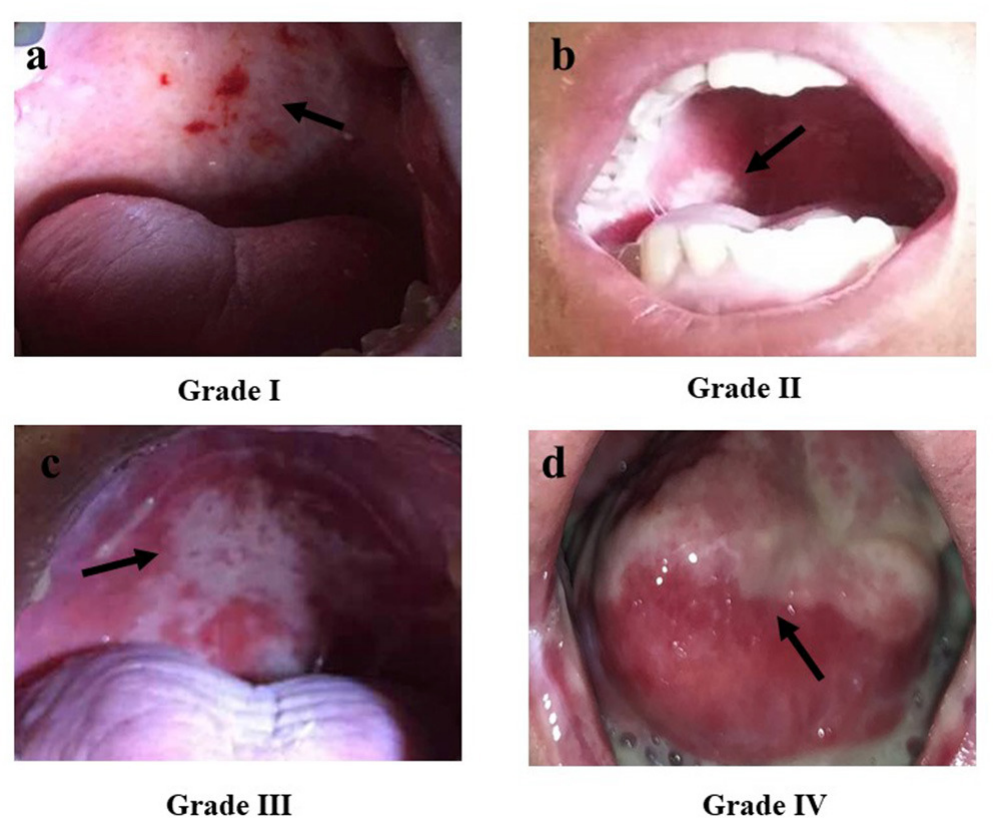
The Radiation Therapy Oncology Group (RTOG) scoring criteria for radiation-induced oral mucositis. **(a)** Grade I: Erythema; **(b)** Grade II: Patchy reaction (<1.5 cm, non-contiguous); **(c)** Grade III: Confluent mucositis (>1.5 cm, contiguous); **(d)** Grade IV: Ulceration, necrosis, bleeding. *Republished with the permission of patients*.

RIOM usually appears 2.5 weeks after RT initiation and continues for 2–3 weeks after treatment completion. Clinically, RIOM is characterized by pain in the oropharynx, dysphagia, language disorders, and nutritional deterioration. Currently, the Radiation Therapy Oncology Group (RTOG) grading is widely used to evaluate the severity of RIOM: Grade I: erythema and mild painful mucositis requiring no analgesics; Grade II: patchy mucositis requiring analgesics; Grade III: confluent mucositis and severe pain requiring narcotic analgesics; and Grade IV: deep ulcerations and/or necrosis (and sometimes bleeding), with extreme pain, and patients cannot eat anymore. Specific examples of different grades of mucositis are shown in Figure 2. A 10–20 Gy dose provokes hyperkeratosis of the oral mucosa, an initial clinical sign accompanied by pain and functional impairment by the 2nd week of treatment (35). At this point, the first signs of erythema are seen. Subsequently, in the third week, once HNC patients have received a dose of more than 20 Gy, they present with a mild and unnoticeable focal area of desquamation (36). In the 4th week, when HNC patients have received more than 30 Gy, diffuse mucosal ulceration appears. Ulcerative lesions are often covered by a pseudomembrane composed of fibrinous exudates and dead cells (37). Many scales can be used to evaluate the severity of mucositis, such as the RTOG scale, World Health Organization oral toxicity scale, Common Terminology Criteria for Adverse Events scale, National Cancer Institute Common Toxicity Criteria (NCI-CTC), and Western Consortium for Cancer Nursing Research stomatitis staging system (Table 1) (38–40).

**Table 1:** Grading criteria.

**Grade**	**1**	**2**	**3**	**4**
RTOG	Erythema	Patchy reaction (<1.5 cm, non-contiguous)	Confluent mucositis (>1.5 cm, contiguous)	Ulceration, necrosis, bleeding
WHO	Sore throat ± erythema, able to eat solid food	Ulcers ± erythema, able to eat solid food	Ulcers with extensive erythema, requires liquid diet	alimentation not possible
CTCAE v5.0	Asymptomatic or mild symptoms; intervention not indicated	Moderate pain; not interfering with oral intake; modified diet indicated	Severe pain; interfering with oral intake	Life-threatening consequences; urgent intervention indicated
NCI-CTC	Erythema of the mucosa	Patchy pseudomembranous reaction (patches generally ≤1.5 cm in diameter and non-contiguous)	Confluent pseudomembranous reaction (contiguous patches generally >1.5 cm in diameter)	Necrosis or deep ulceration; may include bleeding not induced by minor trauma or abrasion
WCCNR	Lesions: none Color: pink Bleeding: none	Lesions: 1–4 Color: slight red Bleeding: none	Lesions: more than 4 Color: moderately red Bleeding: with eating and hygiene	Lesions: coalescing Color: very red Bleeding: spontaneous

*RTOG, Radiation Therapy Oncology Group; WHO, World Health Organization; CTCAE, Common Terminology Criteria for Adverse Events; NCI-CTC, National Cancer institute Common Toxicity Criteria; WCCNR, Western Consortium for Cancer Nursing Research*.

## Symptomatic Treatment of RIOM

The treatment of RIOM is essentially symptomatic, with treatment of complicated infections, and promotion of wound healing of the oral mucosa. If the patient develops severe RIOM, the suspension of RT may be required. The Multinational Association of Supportive Care in Cancer and International Society of Oral Oncology (MASCC/ISOO) Clinical Practice Guidelines are commonly used in the treatment of RIOM (41).

### Pain Management

Pain is the most common symptom among patients with RIOM. Evaluating oral pain is necessary for every patient, and the treatment regime is determined by the level of pain reported by the patients. When RIOM is accompanied by mild pain, acetaminophen and lidocaine can be applied. Acetaminophen with codeine suspension can be used for moderate pain and strong opioids such as morphine or fentanyl need to be used when mucositis progresses to cause severe pain (42). A morphine mouthwash (0.2%) may be effective in treating pain due to RIOM to reduce the need for systemic morphine (39).

### Medications

#### Cytokines and Growth Factors

The destruction of the integrity of oral epithelial cells and the decline in the ability of mucosal repair are major features of RIOM. Many growth factors and cytokines are used clinically to promote mucosal repair and healing in RIOM.

Kannan et al. (43) administered granulocyte macrophage-colony stimulating factor (GM-CSF) to 10 HNC patients. GM-CSF was administered subcutaneously at a dose of 1 μg/kg daily, after a dose of 20 Gy and until the completion of RT. They observed that GM-CSF was able to protect the oral mucosa during RT.

Keratinocyte growth factor (KGF) is a type of human embryonic lung fibroblast growth factor that enhances the regenerative capacity of epithelial tissues and protects them from various toxic agents. Palifermin is an N-terminal, truncated version of KGF. Henke et al. (44) conducted a multicenter, placebo-controlled trial to observe the effect on palifermin in HNC patients suffering RIOM. Patients were randomly assigned to receive weekly palifermin 120 μg/kg or placebo from 3 days before and continuing throughout radiochemotherapy. And they demonstrated that palifermin decreased the duration of RIOM (median, 4.5 vs. 22.0 days) and prolonged the time to develop (median, 45 vs. 32 days) severe RIOM. Le et al. (45) reported a similar trial; HNC patients received palifermin (180 μg/kg) or placebo before starting chemoradiotherapy, once weekly for 7 weeks. The incidence of severe RIOM was significantly lower in the palifermin group than in the placebo group (54 vs. 69%; *P* = 0.041).

Maria et al. (46) conducted a study in an animal model of RIOM, which implanted 5 doses of 2.5 million freshly cultured syngenic aMSCs intraperitoneally, to investigate the ability of mesenchymal stromal cells (MSCs) to repair RIOM. They found that MSCs reduced ulcer duration to 1.6 ± 0.3 days (95% CI 0.0233–3.1 days, a 72% reduction in RIOM ulcer duration). This research initially confirmed the efficacy of MSCs in repairing RIOM, providing a novel treatment regime for RIOM.

Epidermal growth factor (EGF) enhances mucosal wound healing and tissue generation by regulating epithelial cell proliferation, growth, and migration. A placebo-controlled prospective clinical trial assigned HNC patients to a placebo group or to 1 of 3 EGF-treatment groups (10, 50, or 100 microg/mL doses, delivered in a spray, twice daily). This research mainly observed the incidence of severe oral mucositis. Then it revealed that EGF significantly reduced the incidence of severe oral mucositis: 50 μg/mL EGF displayed a 64 response vs. 37% response in the control group (*P* = 0.0246). Therefore, EGF has potential benefits in the treatment of RIOM (47).

In summary, different cytokines or growth factors (KGF, GM-CSF, EGF, and MSCs) affect different cell lines (keratinocytes, macrophages, and fibroblasts), and thus, they promote the healing of the oral mucosa.

#### Anti-inflammatory Agents

RIOM represents an interaction of oral mucosal cells and tissues, proinflammatory cytokines (IL-11, IL-1, and IL-6), and local factors. Benzydamine is a non-steroidal drug that has shown topical anti-inflammatory, analgesic, anesthetic, and antimicrobial activities and can be used to treat RIOM in patients with HNC. Epstein et al. (48) and Sheibani et al. (49) evaluated the efficacy of benzydamine in the treatment of RIOM. Subjects were rinsed with 15 ml benzydamine or placebo daily for 2 min, 4–8 times, before and during radiotherapy and 2 weeks after radiotherapy. Epstein et al. found that phenethylamine significantly (*P* = 0.006) reduced erythema and ulcers by about 30% compared with placebo. Sheibani et al. found that RIOM over grade 3 occurred later in the benzydamine group than in the placebo group.

A multicenter, randomized clinical trial revealed that, when RIOM appeared, 0.1% steroid ointment softens with olive oil was applied to the oral mucosa four times per day, after meals and before bedtime, which can decrease the incidence of grade III RIOM (50). Topically administered corticosteroids have been widely used in the treatment of RIOM, as they can reduce edema, inhibit inflammation, and alleviate symptoms of patients, but the long-term administration of topical steroids may promote candidiasis (51).

Rebamipide is an agent that inhibits the production of inflammatory cytokines, such as IL-1, IL-8, and TNF-α, and may even have anti-ROS effects (52). Yasuda et al. (53) assessed the efficacy and safety of rebamipide in treating RIOM. This gargle solution, at 300 mL per bottle for 1 day, was used in 6 divided doses. The number of patients with severe mucositis (≥ grade 3 RIOM) was higher in the placebo group than in the rebamipide group (83.3 vs. 33.3%, *P* = 0.036), which clearly indicates the contribution of rebamipide in decreasing the severity of oral mucositis.

In addition, honey also has anti-inflammatory effects and can promote wound healing. Khanal et al. (54) conducted a single-blinded, randomized, controlled clinical trial to compare the mucositis-limiting qualities of honey with lignocaine. Each patient would receive an intervention, including 20 ml of either honey or lignocaine gel that would have to be swished about the oral cavity for 2 min and expectorated, for 15 min prior to radiation, 15 min after radiation and once before going to bed. Only 1 of 20 patients in the honey group developed ≥grade III RIOM compared with the lignocaine group, which is 15 of 20 patients. They indicated that honey had a strong protective effect against the development of mucositis.

#### Antimicrobial Agent

RIOM may become infected and require antibiotic therapy. Oral mucosal swabs should be sent for bacterial and fungal culture and drug susceptibility tests to guide the use of antimicrobial agents before treatment (55). Chlorhexidine gluconate is widely recognized as an antimicrobial agent that helps avoid plaque development and control early periodontal infections (56). Stokman et al. (57) observed that the colonization index of Candida and gram-negative bacilli decreased in patients who received active 1 g lozenges containing polymyxin E (2 mg), tobramycin (1.8 mg), and amphotericin B (10 mg) at four times daily during the full course of RT. Therefore, this is an effective way to prevent and treat infections in RIOM.

#### Oral Mucosal Protectant

Misoprostol is a synthetic analog of prostaglandin E1 with anti-inflammatory and mucosa-protecting properties. Veness et al. (58) designed a randomized, double-blind, placebo-controlled trial of misoprostol in patients, but this study was not able to identify a reduction in RIOM in patients receiving misoprostol.

Amifostine has the potential to enable intensified treatment by ameliorating mucosal destruction, but it does not reduce antitumor efficacy. Bourhis et al. (59) conducted a clinical trial, the HNC patients were randomized to receive or not 150 mg/m (2) amifostine, 15–30 min prior to each radiation session. And they found that only 1 patient treated with amifostine developed Grade IV mucositis, compared to 8 patients treated without amifostine. They also suggested that amifostine can reduce the severity and duration of RIOM. Similarly, Veerasarn et al. (60) observed the efficacy of amifostine in the treatment of RIOM and found that amifostine significantly reduced the incidence of grade ≥2 mucositis from 75 to 36%. However, another phase III trial concluded that injecting amifostine every day may result in a high rate of serious adverse effects, leading to the discontinuation of amifostine treatment and sometimes a delay in RT (61).

Receiving oral L-glutamine (L-Gln) is another treatment option for RIOM. A trend toward a beneficial effect on the severity of RIOM was suggested by Huang et al. (62). In a study of 17 HNC patients treated for RIOM with glutamine suspension (16 g glutamine in 240 ml normal saline) or normal saline, all of them received half-mouth irradiation at least. Then, they evaluated the grade of RIOM until 45 Gy/25 fractions, and found that the duration of RIOM ≥ Grade 1 (*p* = 0.0097), Grade 2 (*p* = 0.0232), and Grade 3 (*p* = 0.0168) was shorter in the glutamine arm. they concluded that oral glutamine may shorten the duration and severity of grade III mucositis.

### Low-Level Laser Therapy (LLLT)

LLLT is one of the most recent and promising treatment approaches for RION. MASCC/ISOO recommends LLLT for oral mucositis in HNC patients receiving RT (41). LLLT promotes the proliferation of multiple cells, mainly through the activation of the mitochondrial respiratory chain and initiation of cellular signaling. In addition, it increases the gene expression and protein synthesis of TNF-α, IL-6, and IL-8 to treat RIOM (63). In a study of 39 patients treated for HNC with different protocols of laser phototherapy, the results showed that using a low-power laser alone or in association with a high-power laser when applied three times a week not only maintained RIOM at grades I or II, but also prevented an increase in the nociceptive reaction (64). Maiya et al. (65) treated RIOM patients using a low-level He-Ne laser (wavelength 632.8 nm and output of 10 mW) and found that the mean pain level and mucositis grade were significantly lower in the study group than in the control group (*P* < 0.001). Bensadoun et al. (66) reported a similar study and found that Grade III mucositis occurred in 35.2% of those treated without a low-energy He-Ne laser and 7.6% of those treated with an LEL- 60 mW, wavelength 632.8 nm (*P* < 0.01). These HNC patients received He-Ne laser applications daily for 5 consecutive days (Monday to Friday) each week during the 7 weeks of RT, before the radiation sessions. Moreover, pain relief was significantly better throughout the treatment period (weeks 2–7). In summary, nearly all studies showed good results with reductions in both the incidence and severity of RIOM with no adverse effects, and LLLT can reduce the duration of RIOM to relieve pain. This article summarizes the results of some clinical trials for the treatment of RIOM in Table 2. Most of these studies are comparative experiments and consider the time and incidence of RIOM as the primary endpoint, to judge the efficacy of one or more drugs.

**Table 2:** Clinical trials of various treatments in RIOM.

**No**.	**Reference**	**Sample size**	**Treatment method**	**Evaluation criteria**	**Design**	**Result**
1	Sahebjamee et al. (67)	26	Aloe vera mouthwash vs. benzydamine mouthwash	≥Grade I RIOM development time	RCT, observational	Similar RIOM severity.
2	Sayed et al. (68)	60	Pentoxifylline and vitamin E	≥Grade III RIOM incidence	Prospective, observational	Decreased the duration of RIOM.
3	Bonfili et al. (69)	80	Platelet gel supernatant	≥Grade III RIOM incidence	RCT, observational	Decreased the incidence of WHO grade 3/4 RIOM: 13%
4	Soares et al. (70)	42	LLLT (660 and 808-nm wavelengths vs. only 660-nm wavelength)	≥Grade I RIOM incidence	Parallel, single-blind, two-arm controlled, observational	Group 1 reduced RIOM grade in comparison to Group 2.
5	Huang et al. (71)	71	Oral glutamine vs. placebo	RIOM incidence and severity	Randomized double-blind; Phase III trial	Glutamine had no effect on the severity of RIOM. (*P* = 0.169)
6	Ueno et al. (72)	97	Placebo vs. rebamipide 2% vs. rebamipide 4%	≥Grade III RIOM incidence	Multicenter, randomized, double-blind, placebo-controlled, dose-ranging, phase II trial	The incidences of severe RIOM: 39 vs. 29 vs. 25%.
7	Santos Filho et al. (73)	20	FITOPROT (curcuminoids plus Bidens pilosa Linn)	Adverse reactions development	Phase I trail	FITOPROT was safe and tolerable for RIOM patients.
8	Kawashita et al. (74)	124	Pilocarpine hydrochloride, topical dexamethasone ointment	≥Grade III RIOM incidence	Multicenter, phase II, randomized controlled	Decreased incidence of severe RIOM (*P* = 0.046).
9	Ribeiro da Silva et al. (75)	29	PDT vs. LLLT	The number of clinical cures of RIOM	Open, controlled, and blind, randomized; observational	Satisfactory results in reducing pain.
10	Hadjieva et al. (76)	38	CAM2028-benzydamine	Pain intensity	Observational, Crossover; double-blind; controlled; single-dose; randomized	Relieve pain effectively.
11	Giralt et al. (77)	183	Clonidine vs. placebo	≥Grade III RIOM development time	Phase II, randomized	RIOM developed in 45 vs. 60% (*P* = 0.06)
12	Anderson et al. (78)	223	GC4419 (a superoxide dismutase mimetic)	≥Grade III RIOM development duration	Phase IIb, Randomized, Double-Blind	90 mg produced a reduction of RIOM duration, incidence, and severity.
13	Legouté et al. (79)	97	LLLT	≥Grade III RIOM incidence and time	Phase III	95% of patients exhibited a very good tolerance of LLLT.
14	Sio et al. (80)	275	diphenhydramine-lidocaine-antacid mouthwash	RIOM pain reduction during the 4 h	Phase III, randomized	Deduced pain during the first 4 h after administration.
15	Hua et al. (81)	56	CRO	Total dose of CRO	Observational, prospective	Early introduction of CRO may reduce the total dose of CRO.
16	Jiang et al. (82)	99	Probiotic combination	≥Grade III RIOM incidence	Randomized, double-blind, placebo-controlled	The incidences of grade 3 RIOM was 15.52%.
17	Wu et al. (83)	156	Actovegin	Grade III RIOM incidence and onset time	Multi-center prospective, randomized, multi-center	A low progression rate from grade 2 to 3 (39.2%).
18	Marín-Conde et al. (84)	26	LLLT	RIOM incidence and severity	Prospective randomized controlled	72.7% of the LLLT group showed normal mucosa.
19	Onseng et al. (85)	39	Melatonin vs. placebo	Incidence and time to grade III	Randomized, double-blind, double dummy, placebo-controlled	Incidence of grade 3 RIOM: 42%.
20	Gautam et al. (2)	46	LLLT (λ = 632.8 nm)	RIOM incidence and duration	Double blinded, randomized, lacebo controlled	Reduce the incidence and duration of severe RIOM.

*RIOM, radiation-induced oral mucositis; LLLT, low-level laser therapy; PDT, photodynamic therapy; CRO, controlled-release oxycodone*.

## Preventive Measures

### Oral Care

Oral care is an important integrated prevention strategy for RIOM. The salivary glands do not produce saliva because of radiation damage. The oral cavity gradually becomes acidic, which in turn causes a large number of fungi to multiply. Proper oral care makes the mouth alkaline, reducing the incidence of RIOM. Oral care includes mechanical cleaning (tooth brushing and flossing) and the use of mouthwashes to reduce bacterial aggregation, as well as hydration and lubrication of the oral mucosal surface. It is important to maintain a clean oral cavity through regular brushing, flossing, rinsing, and moisturizing, which can reduce the possibility of oral infection and minimize mucosal tissue injury. Alkalinizing mouthwash is the most frequently used mouthwash for preventing RIOM. The occurrence of RIOM is delayed in HNC patients who undertake continuous oral rinsing for more than 1 month (86). Dodd et al. (87) revealed no significant difference between the efficacy of micronized sucralfate mouthwash and salt and soda mouthwash (86). Alkalinizing mouthwash is the most frequently used mouthwash for preventing RIOM. A randomized controlled trial was conducted to compare the efficacy of an aloe vera mouthwash with that of a benzydamine mouthwash. This revealed that there was no difference between the two groups; thus, an aloe vera mouthwash could be an alternative agent in the prevention of RIOM (67). Kazemian et al. (88) had made a randomized trial that subjects were to rinse with 15 mL benzydamine or placebo for 2 min, 4 times a day from the 1st day of RT to the end, and found that in the benzydamine group, the incidence rate of RIOM grade ≥3 in HNC patients was 43.6%, in contrast to a rate of 78.6% in the placebo group (*P* = 0.001). This trial demonstrated positive effects of benzydamine oral rinse in prevention of RIOM. Saarilahti et al. (89) compared GM-CSF mouthwashes consisting 37.5 microg GM-CSF with sucralfate mouthwashes consisting 1.0 g of sucralfate distilled in water in the prevention of RIOM. This research reported that oral mucositis tended to be less severe in the GM-CSF group (*p* = 0.072), and deduced that the use of GM-CSF mouthwashes may lead to less frequent RT course interruptions from mucositis.

### Nutritional Support

Malnutrition is a common problem among patients with HNC, and 3–52% of HNC patients develop malnutrition without RT (90). During RT, 44% of HNC patients develop malnutrition (91). In addition, 88% of HNC patients develop malnutrition during CCRT (92). Application of local anesthetics before food consumption and using preferably liquid/semisolid foods with high calorie and protein content may be possible approaches (55). Experts have suggested that patients with RIOM should avoid smoking, alcohol, and certain foods, such as tomatoes, citrus fruits, and spicy foods (39). Acidic foods and hot dishes can aggravate RIOM, thus avoiding spicy food can limit any injury to the oral mucosa (39). Positive nutritional support will enhance oral mucosal resistance, reduce the chance of infection, and promote the repair of RIOM. Goda et al. (93) evaluated the efficacy of percutaneous endoscopic gastrostomy (PEG). They suggested that therapeutic PEG is useful for preventing the interruption of RT in HNC patients and should be performed before the radiation therapy dose reaches 30 Gy to avoid severe mucositis. Yamazaki et al. (94) conducted a study and found that the incidence of Grade III or IV oral mucositis was lower in patients receiving PEG than in those who did not receive PEG. In a word, early prophylactic PEG can reduce the occurrence of severe adverse reactions (mucositis and weight loss) and avoid RT interruption. In addition, the timing of nutritional intervention will also affect the incidence and severity of RIOM. A lot of literatures reported that early nutritional intervention would be beneficial to the treatment of HNC patients and reduce the occurrence of adverse reactions. Meng et al. (95) randomly divided a cohort of 78 HNC patients into early (*n* = 46) and late (*n* = 32) nutrition intervention groups. The early group of patients received nutritional support at the beginning of CRT, whereas the late group received such a support until development of the side effects. And they found that the early group showed a lower rate of advanced RIOM (*p* < 0.05). Similarly, Wei et al. (96) made a trial to compare early (*n* = 28) and late (*n* = 26) nutrition intervention groups. The early group received enteral nutrition at the beginning of RT, while the late group received enteral nutrition after restricted feeding. And they clarified that the incidence of high-grade RIOM was significantly lower in the early group than that in the late group (*P* < 0.05). In a word, HNC patients suffer malnutrition early and worsened continuously during RT, so it is important for patients to receive early nutritional support at the beginning of RT, especially in patients at high grade of RIOM (97).

### Radiation Regimen

The severity of RIOM varies with the RT regimen. In recent years, RT technology has become increasingly more advanced for HNC patients with the aim of protecting the oral mucosa, leading to the development of approaches such as IMRT and volumetric modulated arc therapy. Bjarnason et al. (98) compared two groups: morning RT vs. afternoon RT. They revealed a significant reduction in Grade III or greater mucositis in the morning RT group (44.6 vs. 67.3%, *P* = 0.022); morning RT also prolonged the interval until RIOM development (median, >7.9 vs. 5.6 weeks, *P* = 0.033). Dean et al. (99) generated predictive models of severe acute mucositis using RT dose and clinical data. They concluded that receiving intermediate and high doses of oral volume may increase the incidence of mucositis.

### Oral Cryotherapy

Oral cryotherapy offers a convenient and non-invasive prophylactic option for preventing oral mucositis (OM). Riley et al. (100) concluded that oral cryotherapy is effective for the prevention of OM in patients receiving fluorouracil-based chemotherapy. Redding (101) inferred that putting ice chips in the mouth 5 min before administering a 5-FU bolus injection and continuing to do so for 30 min would cool the oral cavity and lead to vasoconstriction. This hypothesis had been certified in a research published in 1991 (102). Patients was randomized divided into two groups, one group received oral cryotherapy during chemotherapy, and the other group served as control group. And they found that OM was reduced by ~50% in the group receiving oral cryotherapy, compared to the control group. Oral cryotherapy is frequently applied in chemotherapy-induced OM, and it has a good preventive effect. But it has not been reported and applied in RIOM. The results of some clinical trials for the prevention of RIOM are summarized in Table 3, excluding retrospective experiments. Most of them consider the incidence and severity of RIOM as the observation standard to judge the effect of different prevention schemes.

**Table 3:** Clinical trials of various preventions in RIOM.

**No**.	**References**	**Sample size**	**Prevention**	**Evaluation criteria**	**Design**	**Result**
1	Chaitanya et al. (103)	60	Rebamipide gargle vs. placevo	RIOM severity	Double blind, randomized	Onset of RIOM: 14.63 d vs. 11.17 d
2	Mantovani et al. (104)	68	GM-CSF	≥Grade III RIOM incidence and duration	Phase II, non-randomized	50% of patients developed RIOM
3	Diaz-Sanchez et al. (105)	7	Bioadhesive chlorhexidine gel 0.2%	Gradation and pain of RIOM	Double-blind, randomized	No clinical improvement
4	Demir Doǧan et al. (106)	80	Black mulberry molasses	Incidence and severity of RIOM	Randomized Controlled	An independent and significant factor. [HR 0.63]
5	Genot-Klastersky et al. (107)	62	LEL	The therapeutic success rate	Prospective	The success rate:81% (95% CI = 61–93%)
6	Elyasi et al. (108)	27	Silymarin (420 mg/d)	Severity of RIOM	Randomized, double-blinded, placebo-controlled	Delayed serious RIOM occurrence.
7	Zanin et al. (109)	72	LLLT (λ = 660 nm)	Grade I-III RIOM incidence and pain	Observational, placebo-controlled	Patients treated with LLLT usually did not present with RIOM or pain.
8	Etiz et al. (110)	44	Oral suspensions of sucralfate	Oral mucosal pain and dysphagia	Prospective, randomized, double-blind, placebo-controlled	Reduced oral pain scores.
9	Gouvêa de Lima et al. (111)	75	LLLT vs. placebo	RIOM severity and the number of RT interruptions	Phase III; randomized; double-blind	Grade 3 or 4 RIOM patients: 4 vs. 5 (Week 2, *p* = 1.0), 4 vs. 12 (Week 4, *p* = 0.08), and 8 vs. 9 (Week 6, *p* = 1.0), respectively.
10	Hamstra et al. (112)	60	Placebo vs. D-met	≥Grade II RIOM incidence	Double-blind placebo-controlled multicenter phase II	Grades 3 to 4 mucositis: 48 vs. 24% (*P* = 0.058)
11	Elkerm and Tawashi (113)	20	DPP	OMAS	Placebo-controlled; observational	Mean oral pain level: 0.7(Day1); 0.07 (Day15); 0 (Day 29)
12	Cheng et al. (114)	42	Oral care	RIOM incidence and pain	Prospective; observational	A 38% reduction in the incidence of ulcerative mucositis.
13	Watanabe et al. (115)	31	Polaprezinc	≤Grade III RIOM incidence and pain	Randomized; observational	Complete plus partial response rate: 88%
14	Giacomelli et al. (116)	40	Orasol Plus (Lapacho-based medication)	≤Grade III RIOM incidence	Phase II	Grade 3: 4 (10%) patients; Grade 4:0
15	Trotti et al. (117)	545	Iseganan HCl (a synthetic peptide)	≥Grade II RIOM incidence	Phase III; multinational, randomized, double-blind, controlled	9% of the patients did not develop ulcerative OM (Grades 2, 3, 4) (*p* = 0.998)
16	Zhu et al. (118)	20	Epigallocatechin-3-gallate (EGCG)	Safety of EGCG	Phase I; prospective, non-randomized,	No patients experienced ≥Grade III RIOM; the recommended dose of EGCG is 1,760 μmol/L.

*RIOM, radiation-induced oral mucositis; GM-CSF, granulocyte-macrophage colony stimulating factor; HR, hazard ratio; CI, confidence interval; CCRT, concurrent chemoradiation therapy; LLLT, low-level laser therapy; Low-energy laser, LEL; D-met; D-methionine; DPP, date palm pollen; OMAS, Oral Mucositis Assessment Scale*.

## Discussion

RIOM is a common complication in patients with HNC after RT. The main clinical symptoms are oral pain, mucosal ulcers, and dysphagia. Currently, there are numerous prevention and treatment strategies for RIOM. Good oral health, adequate nutritional support, and advanced RT approaches can prevent RIOM. In addition, RIOM treatment focuses on reducing symptoms and complications. The treatment regimens include analgesic and anti-inflammatory drugs, medications and LLLT. For grade I and II RIOM, the treatments are mainly concerned with oral care, especially postprandial oral cleaning, mouthwash with saline and nutritional support. Apart from those, patients can also use mucosal protective agents. For grade III-IV RIOM, in addition to the treatment measures of grade I-II, patients can also add anti-inflammatory drugs and hormones. And we need to pay attention to the management of pain, adding different analgesics according to the level of pain. In addition, LLLT can also be considered to use for patients. However, at present, for the prevention and treatment of radiation-induced mucosal injury, the medical community has not yet formed any standardized medical nutrition treatment program, and the mechanism of mucosal injury remains to be thoroughly studied.

## Author Contributions

XJ and LD: conceptualization. QZ and ZL: software and investigation. ZZ: resources. SL and QZ: writing-original draft preparation. LM, LD, and XJ: writing-review and editing. XJ: funding acquisition. All authors read and approved the manuscript.

## Conflict of Interest

The authors declare that the research was conducted in the absence of any commercial or financial relationships that could be construed as a potential conflict of interest.

## Abbreviations

SCCRT: concurrent chemoradiation therapy
CTCAE: Common Terminology Criteria for Adverse Events
GM-CSF: granulocyte macrophage-colony stimulating factor
HNC: head and neck cancer
IL-1β: Interleukin-1β
IL-6: Interleukin-6
IMRT: intensity-modulated RT
LEL: low-energy helium-neon laser
L-GLN: L-glutamine
LLLT: low-level laser therapy
MASCC/ISOO: Multinational Association of Supportive Care in Cancer and International Society of Oral Oncology
MMP: matrix metalloproteinase
NCI-CTC: National Cancer institute Common Toxicity Criteria
PBRT: proton beam radiation therapy
PEG: percutaneous endoscopic gastrostomy
RIOM: Radiation-induced oral mucositis
RT: radiotherapy
RTOG: Radiation Therapy Oncology Group
SBRT: stereotactic body radiation therapy
TNF-α: tumor necrosis factor-α
VMAT: volumetric modulated arc therapy
WCCNR: Western Consortium for Cancer Nursing Research
WHO: World Health Organization.
